# Insect Odorant Receptors: From Structure and Evolution to Mechanism and Application

**DOI:** 10.3390/insects17050496

**Published:** 2026-05-13

**Authors:** Jinfeng Hua, Huifeng Li, Yongmei Huang, Yanqing Li, Zhenwei Li, Tianyuan Chen, Chao Pan, Renbing Qin, Yongbo Wang

**Affiliations:** 1 Department of Sweet Potato Genetic Breeding and Application, Institute of Maize Research, Guangxi Academy of Agricultural Sciences, Nanning 530007, China; 605lifeng@163.com (H.L.); huangyongmei322@163.com (Y.H.); liyq2004.7@163.com (Y.L.); lizhenweihhh@163.com (Z.L.); tianyuanchen@126.com (T.C.); 2 JSNU-UWM International Cooperation Joint Research Laboratory of Food Safety and Microbial Functional Genomics, School of Life Science, Jiangsu Normal University, Xuzhou 221116, China; chaopan@jsnu.edu.cn; 3 School of Food Science, Henan Institute of Science and Technology, Xinxiang 453003, China; qinrenbing86@hist.edu.cn; 4 Department of Cotton Breeding Laboratory, Institute of Cotton and Sericulture, Hunan Academy of Agricultural Sciences, Changsha 415101, China; wangyongbo040923@163.com

**Keywords:** insect olfaction, odorant receptor, Orco, cryo–EM, signal transduction, molecular evolution, reverse chemical ecology, structure–function relationship

## Abstract

Insect odorant receptors (ORs) are pivotal molecular interfaces that translate environmental chemical cues into neuronal electrical impulses, thereby governing essential behaviors such as foraging, mating, oviposition, and predator avoidance. The past three years have witnessed a paradigm shift driven by high-resolution cryo-electron microscopy (cryo–EM) structures of OR-odorant receptor co-receptor (Orco) heterocomplexes, which definitively established the 1:3 stoichiometry (one odorant-specific OR subunit and three Orco subunits) of the functional ion channel. These structures have revealed the architecture of the ligand-binding pocket and the conformational dynamics underlying channel gating. This structural framework has illuminated long-standing questions regarding the evolution of ORs from ancestral gustatory receptors and their lineage-specific expansion via a “birth-and-death” model, enabling adaptation to diverse ecological niches. Concurrently, the long-debated signal transduction mechanism has been reconciled by evidence of a unified bimodal system, where OR–Orco complexes function as both direct ligand-gated ion channels and activators of an IP3-dependent metabotropic cascade. Here, we integrate these recent breakthroughs—from atomic-level structures and evolutionary genomics to in vivo functional validation—with classical knowledge of OR expression, localization, and diversity. We further synthesize the emerging field of structure-guided applications, including virtual screening for novel semiochemicals and the development of RNAi- and CRISPR-based strategies for pest management. This comprehensive review provides a framework for understanding the molecular logic of insect olfaction and its exploitation for biotechnological innovation.

## Simple Summary

Insects possess an extraordinary sense of smell, which is crucial for finding food, mates, and suitable habitats. This remarkable ability is mediated by odorant receptors (ORs) located on their antennae and other olfactory organs, such as maxillary palps. Recent technological advances, particularly in cryo-electron microscopy, have allowed scientists to visualize the three-dimensional structure of these receptors for the first time. These atomic-level images reveal how ORs capture odor molecules and convert them into electrical signals sent to the brain. This review synthesizes these structural breakthroughs with new insights into how ORs have evolved across different insect species to detect specific chemical landscapes. We further explore how this molecular knowledge is being translated into practical applications, such as designing environmentally friendly pest control strategies that disrupt insect communication, offering sustainable solutions for agriculture.

## 1. Introduction

The evolutionary success of insects, the most speciose group of animals, is inextricably linked to the sensitivity and specificity of their olfactory systems [1,2]. From the earliest observations of Jean-Henri Fabre to modern genomic analyses, the ability of insects to decode complex chemical signals has captivated biologists [3,4]. This olfactory capability underpins critical behaviors including locating food sources, identifying suitable oviposition sites, recognizing conspecifics for mating, and avoiding natural enemies [5,6].

The insect olfactory pathway represents a sophisticated signal transduction cascade. Volatile odorant molecules penetrate pores in the sensilla—cuticular structures housing the olfactory system—and are transported through the aqueous sensillar lymph by odorant-binding proteins (OBPs) and chemosensory proteins (CSPs) [7,8]. These OBP-odorant complexes are delivered to the dendritic membrane of olfactory sensory neurons (OSNs), where they interact with odorant receptors (ORs) [9,10]. OR activation converts the chemical signal into an electrical one, which is processed in the antennal lobe before being relayed to higher brain centers to drive behavior [11,12].

The molecular era of OR research began at the turn of the millennium with the discovery of the first insect OR genes in *Drosophila melanogaster* [13,14]. Subsequent years saw an explosion in the identification of OR repertoires across hundreds of insect species, revealing a superfamily of seven-transmembrane-domain proteins with a topology inverse to that of vertebrate G-protein-coupled receptors (GPCRs) [15,16]. However, a true paradigm shift has occurred in the last three years, driven by cryo-electron microscopy (cryo-EM) structures of OR-Orco complexes from multiple species [17,18]. For the first time, we can visualize the atomic architecture of the receptor, its ligand-binding pocket, and the ion channel pore. This structural insight, combined with the recent elucidation of a complete IP3-mediated signaling pathway [19], has fundamentally reshaped our understanding of how these receptors function.

This review aims to synthesize these transformative discoveries with the broader context of OR biology. We will explore the structural blueprint of ORs, their evolutionary history and patterns of diversification, the newly unified model of signal transduction, and the burgeoning field of structure-guided applications, offering a comprehensive view of insect ORs from molecule to organism.

## 2. The Structural Blueprint of Insect Odorant Receptors

### 2.1. Topology and Classification: A Unique Membrane Protein Fold

Insect ORs are hydrophobic membrane proteins, typically 300–600 amino acids in length, residing on the dendritic membranes of OSNs [20]. Initial bioinformatic analyses predicted a seven-transmembrane (7-TM) domain structure, leading to their initial misclassification as GPCRs. However, a defining feature of insect ORs is their inverted topology compared to canonical GPCRs: the N-terminus is intracellular, and the C-terminus is extracellular [21,22]. This unique fold places them within the structural superfamily of ion channels rather than GPCRs [23].

Functionally, ORs are divided into two distinct classes: Tuning odorant receptors (ORs): This large, divergent family is responsible for the specific detection of a vast array of volatile chemicals. It includes “general” ORs that detect plant volatiles and pheromone receptors (PRs) tuned to conspecific sex pheromones [24]. The odorant receptor co-receptor (Orco): In stark contrast to the diversity of ORs, Orco is a highly conserved, single-copy gene in virtually all insects [25,26]. Orco does not bind odorants itself but acts as an obligate ion channel-forming partner for all tuning ORs. While OR sequences can share as little as 20% identity across species, Orco orthologs are typically >70% identical, underscoring its essential and conserved structural role [27,28].

Given its essential role, Orco deserves special attention. Orco is a highly conserved protein that does not bind odorants directly but forms the structural scaffold and ion-conducting pore of the functional complex. In this homomeric state, the S7b helices line the central pore. In the heteromeric OR-Orco complex, the three Orco subunits maintain this structural role, with their S7b helices contributing to the pore. Mutagenesis studies have identified a conserved “ionic lock” motif (R/KxxxxE/D) in Orco that is critical for keeping the channel closed in the absence of odorant binding to the tuning OR [29].

### 2.2. The 2024–2025 Revolution: High-Resolution Cryo-EM Structures

The years 2024 and 2025 marked a watershed moment, with multiple groups independently solving the high-resolution structures of OR-Orco complexes, providing the first atomic-level views of these elusive proteins.

Wang et al. [17] reported the cryo-EM structure of the alarm pheromone receptor ApOR5-Orco from the pea aphid, *Acyrthosiphon pisum*, in both ligand-free and ligand-bound states at resolutions of 2.8–3.2 Å. This structure definitively revealed that the functional unit is an obligate heterotetramer composed of one tuning OR subunit and three Orco subunits (1:3 stoichiometry), although minor states such as 2:2 were also observed. The tuning OR subunit contains the sole ligand-binding site, while the three Orco subunits act as a structural scaffold. Binding of the alarm pheromone (E)-β-farnesene to ApOR5 induces a conformational change, notably an outward movement of its pore-lining helix, S7b, creating an asymmetric opening for ion flow.

Independently, Zhao et al. [18] published the structures of OR-Orco complexes from the yellow fever mosquito, *Aedes aegypti* (AaOR10-Orco), and the malaria mosquito, *Anopheles gambiae* (AgOR28-Orco), confirming the 1:3 stoichiometry. These structures were complemented by work on the *Drosophila* OR22a-Orco complex [19]. The congruence of these structures from evolutionarily distant insects (fly, mosquito, aphid) points to a universal architectural principle.

**Figure 1:**
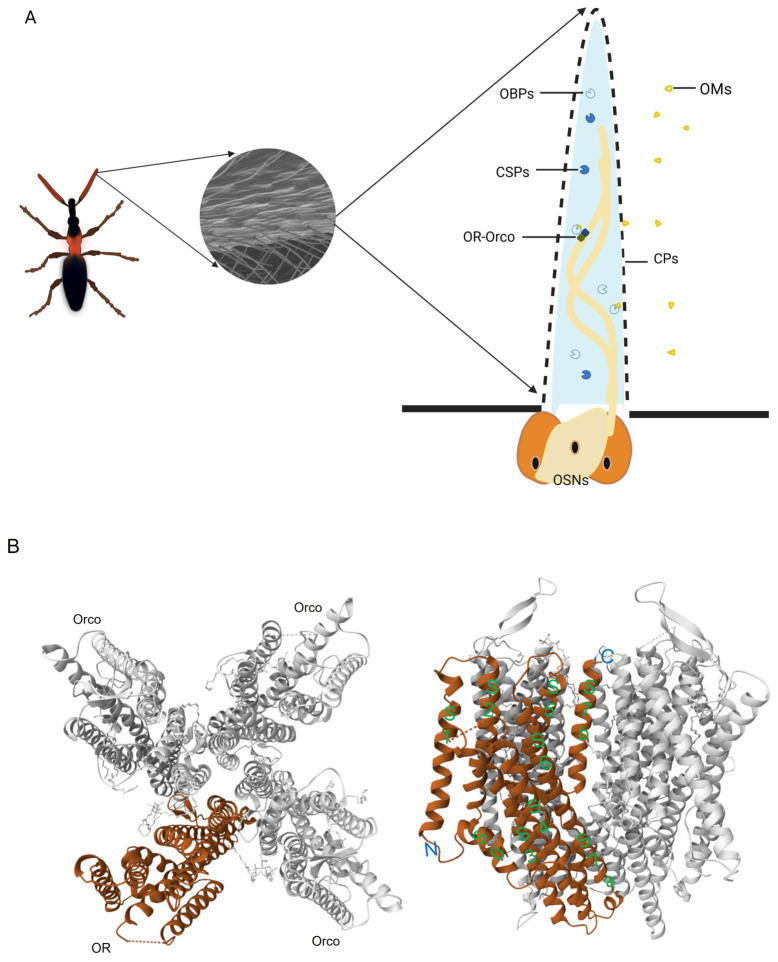

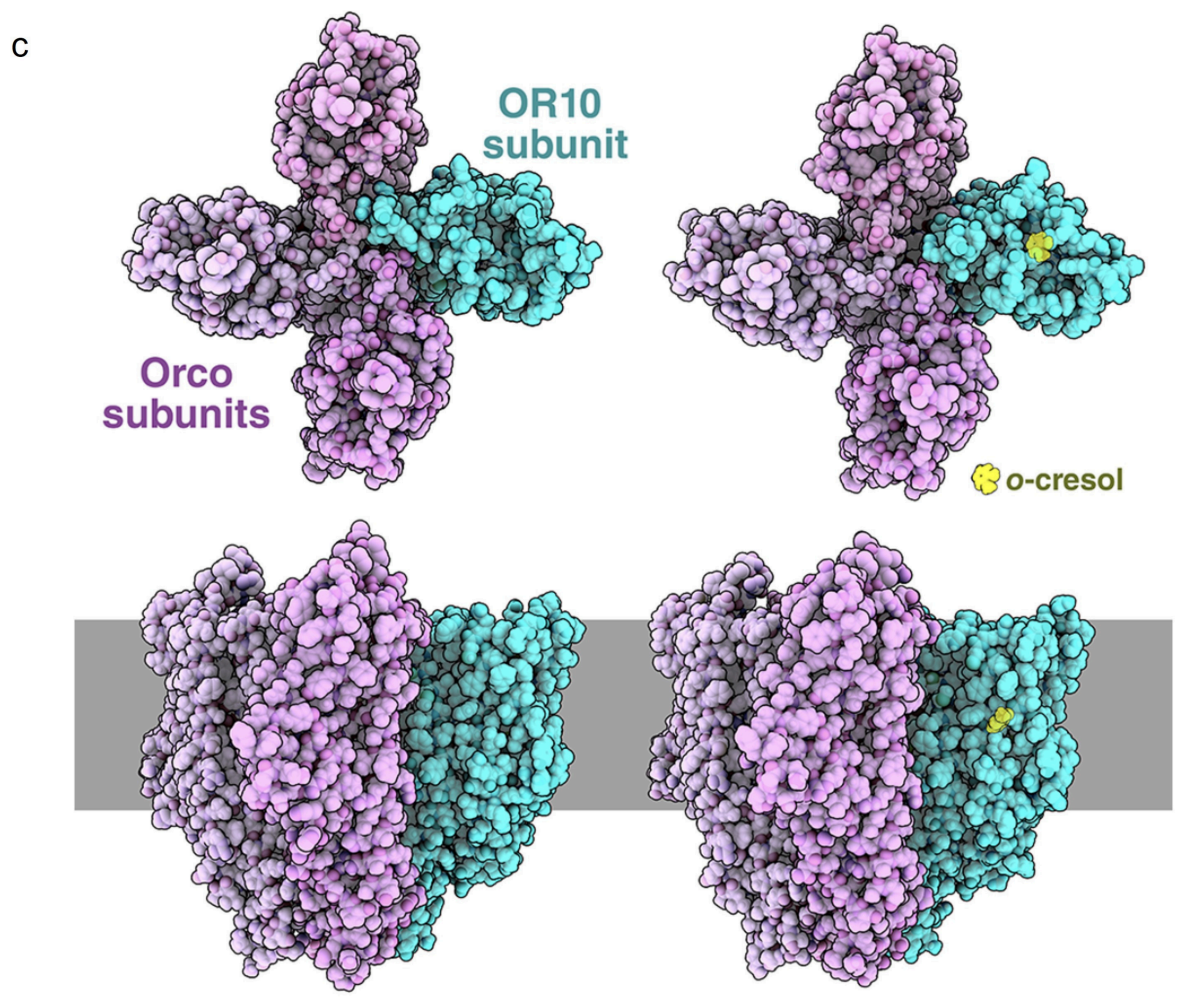
Structural architecture of the insect olfactory system and OR-Orco complex. (**A**) Schematic diagram of the insect olfactory pathway. Odorant molecules (OMs) enter the sensillum through cuticular pores and are transported by odorant-binding proteins (OBPs) and chemosensory proteins (CSPs) across the sensillar lymph to reach the OR-Orco complexes on the dendritic membrane of olfactory sensory neurons (OSNs). (**B**) Three-dimensional cryo-EM structure of the ApOR5-Orco heterocomplex. The membrane topology of insect ORs (intracellular N-terminus, extracellular C-terminus) is shown. Key features are labeled: S0–S7 (green). (**C**) Three-dimensional cryo-EM structure of the AaOR10-Orco heterocomplex. The complex forms a heterotetrameric ion channel with a predominant 1:3 stoichiometry (one tuning OR subunit in teal, three Orco subunits in pink). On the right, the open receptor is shown binding to a ligand, o-cresol (shown in yellow).

These structural studies have revealed conserved features (Figure 1). Subunit topology: Each subunit contains seven transmembrane helices (S1–S7) and a short intracellular helix (S0). Tetramer assembly with 1:3 stoichiometry: Four subunits (one tuning OR and three Orco) are arranged in a windmill shape, forming a central ion-conducting pore. Ligand-binding pocket: Located between S2–S6 of the tuning OR subunit, facing outward, composed of variable hydrophobic residues and hydrogen-bond donors, dictating specificity. For example, in ApOR5, residues F192, Y206, and W209 form a hydrophobic cage that coordinates (E)-β-farnesene. Ion-channel gating: Coordinated rotation of S5–S7b helices drives channel opening, with S7b as the pore-lining helix. The S7b helix of the tuning OR subunit undergoes a 15° outward rotation upon ligand binding. Subunit interface (Anchor Domain): Concentrated in an anchor domain formed by intracellular parts of S4, S5, S6, and S7a. This domain is critical for the stable assembly of the heterotetramer.

Molecular dynamics simulations have further validated the functional significance of the 1:3 stoichiometry. Zhang et al. [30], using the locust aggregation pheromone receptor LmOR35-Orco as a model, demonstrated that the 1:3 assembly is the most energetically favorable and functionally efficient, exhibiting optimal channel opening and ion conduction.

## 3. Molecular Evolution and Diversity of the OR Repertoire

### 3.1. Evolutionary Origins: From Taste to Smell

The availability of genomes from across the insect phylogeny has allowed for robust reconstruction of OR evolution. It is now widely accepted that ORs evolved from the more ancient gustatory receptor (GR) family, which primarily detects non-volatile tastants [1,31]. This evolutionary transition is estimated to have occurred around 300 million years ago, coinciding with the adaptation of insects to flight and the increased importance of detecting volatile cues over long distances [32,33].

Recent structural biology has provided remarkable support for this evolutionary relationship. The cryo-EM structure of a GR from *Drosophila melanogaster* (DmGR43a) revealed a tetrameric architecture strikingly similar to that of ORs, with the same inverted 7-TM topology and a central pore lined by S7b helices [34]. This structural conservation suggests that the basic ion channel architecture predates the functional divergence of GRs and ORs. The transition from GR to OR likely involved changes in the ligand-binding pocket that shifted specificity from non-volatile sugars and bitter compounds to volatile odorants. This structural insight provides a powerful framework for understanding how a new sensory modality evolved through modification of an existing protein scaffold.

After their divergence from GRs, ORs underwent massive lineage-specific expansions and diversification. Interestingly, Orco itself appears to have arisen from within the OR family later in evolution. In some basal, wingless insects like the jumping bristletail *Machilis hrabei*, functional ORs can form homotetrameric channels independently of Orco [35]. For example, the homomeric Orco channel from the jumping bristletail *Machilis hrabei* (MhOR5) exhibited a tetrameric architecture, suggesting that ancestral ORs could function independently before the emergence of the obligate OR-Orco heterocomplex [36]. This suggests an evolutionary trajectory where ancestral ORs functioned alone, and the specialized, highly conserved Orco subunit was subsequently co-opted to form the more efficient and stable heteromeric complexes seen in all winged insects (Pterygota) [37].

### 3.2. Dynamic Evolution and the “Birth-And-Death” Model

The size of the OR gene family varies dramatically across insect orders, reflecting adaptations to specific ecological niches [38,39]. Family sizes range from as few as 5 in some primitive insects to over 500 in social insects like ants and bees, which require sophisticated communication systems [40,41]. Table 1 summarizes the *OR* gene counts and characteristics for a representative set of insect species across major orders, with a focus on recent high-quality studies.

This diversity is generated and maintained by a “birth-and-death” model of evolution [61]. Tandem gene duplications create new *OR* gene copies, which accumulate mutations. These paralogs may be retained if they acquire a new beneficial function (neofunctionalization) or are lost (pseudogenization) [62]. A clear example of this process is seen in the red flour beetle (*Tribolium castaneum*), where a recent expansion of OR genes on chromosome 3 is associated with the detection of stored-product volatiles [49]. This process allows insect lineages to rapidly adapt their olfactory capabilities to new hosts, habitats, and social structures. Within an OR repertoire, pheromone receptors (PRs) often show signatures of positive selection, particularly in residues lining the ligand-binding pocket, driving reproductive isolation and speciation [63,64].

### 3.3. Structural Conservation Amidst Sequence Divergence

Despite extreme sequence divergence between tuning ORs, their three-dimensional structures are remarkably conserved. Using AlphaFold2 to predict structures of deorphanized ORs, Li et al. [65] demonstrated that functionally critical residues consistently map to two key structural hotspots: the ligand-binding pocket (variable, explaining specificity) and the ion channel gate (conserved, ensuring universal function). This “variable pocket, conserved scaffold” principle elegantly explains how evolution tinkers with ligand specificity while preserving core signaling machinery [66].

## 4. Reconciling the Signal Transduction Mechanism

### 4.1. Ionotropic Gating: The Fast Pathway

**Figure 2:**
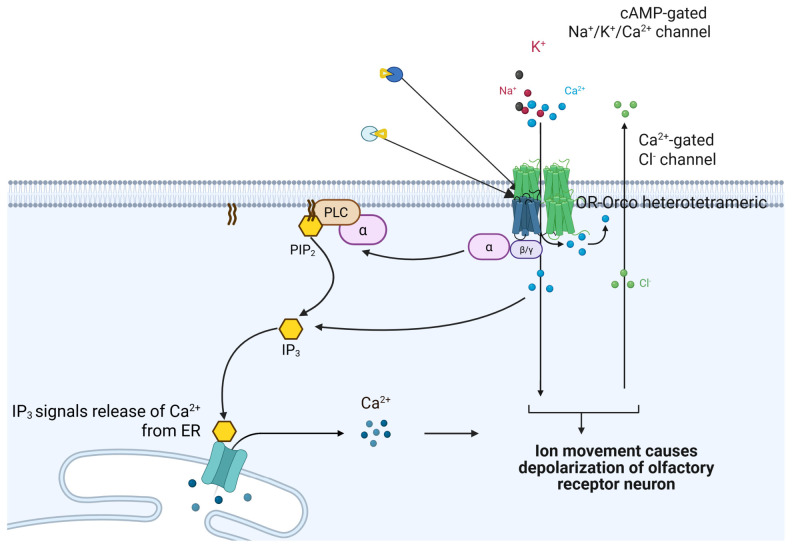
A unified bimodal model for insect OR signal transduction. Ionotropic pathway: Odorant binding directly opens the OR-Orco channel, allowing cation influx and rapid membrane depolarization. Metabotropic pathway: OR activation couples to PLC via Clvs2, hydrolyzing PIP2 to IP3 and DAG. IP3 triggers Ca^2+^ release from intracellular stores (e.g., ER), amplifying the signal. Integration: The rapid ionotropic response provides initial speed, while the IP3 cascade provides sensitivity and amplification, together ensuring robust olfactory coding.

The high-resolution structures have provided an atomic-level view of the ion channel gating mechanism, which can be summarized as follows [17,18,30]: Ligand binding: Odorant molecules enter the binding pocket of the tuning OR subunit, forming hydrophobic interactions and hydrogen bonds with key residues. In ApOR5, the (E)-β-farnesene molecule is stabilized by π-alkyl interactions with F192 and Y206. Local conformational changes: Ligand binding triggers subtle adjustments in helices S2–S6, particularly displacement of helix S4. This displacement is transmitted via a conserved “transmission interface” involving residues on S4 and S5. Cooperative rotation: Conformational changes propagate through subunit interfaces to the S7b helix of the tuning OR subunit, causing a ~15° outward rotation and outward movement. Channel opening: Cooperative movement of the four S7b helices (one from the tuning OR and three from Orco) expands the central pore diameter from ~3 Å in the closed state to ~8 Å in the open state, allowing passage of Na^+^, K^+^, and Ca^2+^. Signal termination: After ligand dissociation or degradation, the channel reverts to its closed conformation. A conserved “ionic lock” motif (R/KxxxxE/D) in the S7b helix of each subunit impedes ion flow in the resting state; ligand binding triggers helical rotation that disrupts this lock, enabling channel opening [29]. Figure 2 shows a unified bimodal model for insect OR signal transduction. Ionotropic pathway (fast): Odorant binding directly opens the OR-Orco channel, allowing cation influx (Na^+^, K^+^, Ca^2+^) and rapid membrane depolarization. Metabotropic pathway (slow, amplifying): OR activation couples to phospholipase C (PLC) via the Clvs2 protein, hydrolyzing PIP2 to IP3 and DAG. IP3 triggers Ca^2+^ release from intracellular stores (e.g., endoplasmic reticulum, ER), amplifying the signal. Integration: The rapid ionotropic response provides initial speed, while the IP3 cascade provides sensitivity and amplification, together ensuring robust olfactory coding (abbreviations: PLC, phospholipase C; PIP2, phosphatidylinositol 4,5-bisphosphate; IP3, inositol trisphosphate; DAG, diacylglycerol; ER, endoplasmic reticulum).

### 4.2. Signal Termination

Signal termination is critical for temporal resolution. This is primarily achieved by odorant-degrading enzymes (ODEs) in the sensillar lymph, which rapidly metabolize odorants [67,68]. Sensory neuron membrane proteins (SNMPs), initially thought to be solely involved in pheromone transport, have also been implicated in signal clearance by aiding the removal of spent OBP-odorant complexes [69,70]. Recent studies have identified specific esterases and cytochrome P450s as key ODEs in moths and mosquitoes [71,72].

## 5. Functional Landscapes and Emerging Applications

### 5.1. Functional Diversity of Tuning ORs

Table 2 summarizes the expression patterns of selected OR genes across different tissues and developmental stages, illustrating the dynamic and tissue-specific regulation of the insect olfactory system. The functional characterization (“deorphanization”) of individual ORs has revealed a vast landscape of ligand-receptor interactions governing specific behaviors [44,73]. It is important to note that deorphanization identifies a receptor’s molecular tuning, but linking this to behavior requires additional in vivo studies.

General Odorant Receptors: These detect host plant volatiles and environmental cues. For instance, *HassOR67* in *Helicoverpa assulta* recognizes nonanal, driving oviposition site selection [81]. *MpOR3c* in *Myzus persicae* mediates nonanal detection, guiding it to host plants [59]. A recent meta-analysis by Comte et al. [82] found that over 60% of deorphanized ORs in Lepidoptera respond to green leaf volatiles, highlighting the importance of these cues for host finding.

Pheromone Receptors (PRs): PRs are exquisitely tuned to conspecific sex pheromones. In *Cnaphalocrocis medinalis*, *CmedPR1* responds to major pheromone components [83]. PRs represent a highly specialized subfamily that has undergone rapid evolution. In moths, two main PR lineages have been identified: the classical PRs that detect type-I pheromones (long-chain unsaturated alcohols, aldehydes, and acetates) and a more recently discovered lineage (PR-like) that detects type-II pheromones (polyunsaturated hydrocarbons and epoxides) [63]. A landmark discovery by Zhang et al. [42] showed that *HarmOR56*, a female-specific OR in *Helicoverpa armigera*, mediates oviposition deterrence by recognizing methyl esters signaling an over-exploited host, representing a sophisticated “push” signal.

### 5.2. The Pivotal Role of Orco in Olfactory Signal Transduction

Orco is the indispensable partner for all tuning ORs. Beyond its structural role, it is essential for:

1. Correct Subcellular Localization: Orco is required for the proper trafficking and stabilization of tuning ORs on the OSN dendritic membrane [84].
2. Forming the Ion Channel Pore: Orco S7b helices are primary constituents of the pore [17].
3. Enhancing Sensitivity: Orco dramatically increases the sensitivity of the OR complex to its ligand [28].

Disrupting Orco function leads to profound olfactory deficits. RNAi-mediated knockdown of Orco in *Chrysopa pallens* and *Callosobruchus maculatus* significantly reduces electrophysiological responses to odorants and pheromones [72,85]. CRISPR-Cas9 knockout of Orco has been achieved in multiple species, including *Drosophila*, *Tribolium castaneum*, *Harpegnathos saltator*, and *Spodoptera frugiperda*. In all cases, Orco knockout resulted in severe olfactory deficits, disrupted mating behavior, and reduced fitness, validating Orco as a promising target for genetic pest control [86,87].

### 5.3. Structure-Guided Applications in Pest Management

The availability of high-resolution OR structures has opened new frontiers in applied entomology, moving beyond trial-and-error to rational, structure-based design [88,89]. Reverse Chemical Ecology: This approach uses OR sequences and predicted structures to screen for cognate ligands in silico or in vitro, accelerating the discovery of behaviorally active compounds [82,90]. Virtual Screening for Novel Behavior Modifiers: Using the 3D structure of an OR’s binding pocket for virtual screening recently identified 2-ethylbenzofuranone, which activated ORs from 14 lepidopteran species and outperformed traditional sex pheromones in field trials by attracting a broader range of species [65,91]. Similarly, in silico screening targeting a mosquito OR2 led to pyrethroid derivatives with increased repellency [31,92]. These successes demonstrate the power of structure-guided approaches, although challenges remain in predicting how these compounds will perform in complex natural environments. Genetic Pest Control Strategies: Precise gene-editing tools like CRISPR-Cas9 are being used to validate OR function and explore population control. Knockout of HarmOR56 abolished oviposition deterrence in *H. armigera*, demonstrating a potential “push” strategy [42]. RNAi targeting Orco remains a promising tool for species-specific olfactory disruption, but its field application is currently limited by issues of stability and delivery [65,87].

## 6. Future Perspectives

The last three years have fundamentally redefined our understanding of insect ORs. Future research will build on this foundation:

1. Capturing Conformational Dynamics: Time-resolved cryo-EM and advanced molecular dynamics simulations will visualize the complete movie of channel gating [93].
2. Predicting Function from Structure: Leveraging AlphaFold2 and AI to predict structures of thousands of uncharacterized ORs will accelerate deorphanization and enable cross-species functional predictions [65,94,95].
3. Understanding Combinatorial Coding: The challenge to the “one neuron, one receptor” model by evidence of co-expression of ORs, IRs, and GRs in single OSNs requires investigation of how the brain integrates combinatorial inputs [96,97].
4. Designing Next-Generation Semiochemicals: Combining high-throughput virtual screening, structure-guided design, and olfactory coding insights will enable the development of exquisitely specific “next-gen” semiochemicals (super-agonists, antagonists) for sustainable pest management [91,98].
5. Integrating Olfaction with Other Modalities: Future research should explore how olfactory input integrates with vision, taste, and mechanosensation to generate context-appropriate behaviors [99,100,101].

## 7. Conclusions

Research on insect odorant receptors has traversed a remarkable path from gene discovery to structural revelation and functional integration. The recent cryo-EM structures have provided a long-awaited molecular blueprint, revealing the conserved architecture and dynamic gating of the OR-Orco ion channel. This structural insight has illuminated our understanding of their evolutionary history and functional diversification. The resolution of the signal transduction controversy with the discovery of a unified bimodal ionotropic and IP3-mediated metabotropic pathway adds a new layer of physiological complexity. Together, these advances are not only deepening our fundamental understanding of how insects perceive their world but are also providing the molecular tools to manipulate that perception for sustainable pest management, marking a new era in the field of insect olfaction.

## Figures and Tables

**Table 1:** Comparative Analysis of Odorant Receptor Gene Repertoires in Representative Insect Species (2020–2026).

Order	Representative Species	No. of Deorphanized ORs	Major Ligand Classes	Key Behavioral Functions	Selected References
Lepidoptera	*Helicoverpa armigera*	>20	Sex pheromones, green leaf volatiles, terpenoids	Oviposition deterrence, mating, host finding	[42,43,44]
Lepidoptera	*Spodoptera frugiperda*	~15	Pheromones, plant volatiles (e.g., (Z)-9-tetradecenyl acetate)	Oviposition preference, male attraction	[45,46]
Lepidoptera	*Bombyx mori*	4 (classic)	Bombykol, bombykal	Mating	[47,48]
Diptera	*Drosophila melanogaster*	>50	Fruit esters, alcohols, acids	Food search, courtship	[13,49,50]
Diptera	*Aedes aegypti*	~12	Indole, ammonia derivatives, octenol	Host seeking, ovip	[18,51]
Diptera	*Anopheles gambiae*	~10	Human skin odors, plant volatiles	Host preference	[18,52]
Coleoptera	*Tribolium castaneum*	~8	Pheromones (4,8-dimethyldecanal), food odors	Aggregation, mate recognition	[53]
Coleoptera	*Cylas formicarius*	6 (via OBPs/CSPs)	Host plant volatiles (e.g., (E)-2-hexenal)	Host location	[6,54,55]
Hymenoptera	*Harpegnathos saltator*	~9	Cuticular hydrocarbons, general odorants	Nestmate recognition, social behavior	[41,56]
Hymenoptera	*Apis mellifera*	~12	Queen pheromone components (9-ODA), floral scents	Queen retinue, foraging	[57]
Hemiptera	*Acyrthosiphon pisum*	5	Alarm pheromone (E)-β-farnesene	Escape behavior	[17,58]
Hemiptera	*Myzus persicae*	4	Green leaf volatiles (e.g., nonanal)	Host plant selection	[59]
Orthoptera	*Locusta migratoria*	3	Aggregation pheromone (4-vinylanisole), plant volatiles	Aggregation, feeding	[19,30,60]

**Table 2:** Tissue- and Stage-Specific Expression Patterns of Selected Odorant Receptor Genes in Insects (2020–2026).

Species	Genes	Expression Pattern	Key Finding	Reference
*Hyphantria cunea*	*HcunORs (21 genes)*	Antennae-high	Most *ORs* antennae-high	[74]
*Clostera restitura*	*CresOR37,41,43,56*	Male antennae-specific	Sex-biased expression suggests roles in pheromone detection	[75]
*Apriona germari*	*AgerOR1,3,4,37,40*	Female antennae-specific	Sex- and tissue-specific expression; *AgerOR9* in female maxillary palp	[76]
*Drosophila suzukii*	*DsuzOrco*	3rd instar larvae (trace); adult high	Developmental regulation; increases with age	[68]
*Locusta migratoria*	*LmigOR12,20*	Antennae = palps; palps-high	Distinct spatial expression patterns in olfactory organs	[60]
*Chrysopa pallens*	*CpalOR3,* etc. *(28 genes)*	Antennae-high	Broad tissue distribution; *CpalOR6* in thorax; *CpalOR26,27* in abdomen	[77]
*Galeruca daurica*	*GdauOR4, Orco*	Antennae-highest	Sex- and tissue-biased expression; *GdauOR1* female antennae-high	[78]
*Eupeodes corollae*	*EcorOR4*	Antennae-specific	Strict antennae-specific expression; conserved recognition of 1-octen-3-ol	[79]
*Callosobruchus chinensis*	*CchiOR8, 10*	Antennae-high; female antennae-high	Sexually dimorphic expression; spatio-temporal expression profiling	[80]

## Data Availability

No new data were created or analyzed in this study. Data sharing is not applicable to this article.
